# Identification of potential genes for human ischemic cardiomyopathy based on RNA-Seq data

**DOI:** 10.18632/oncotarget.13331

**Published:** 2016-11-12

**Authors:** Wan Li, Liansheng Li, Shiying Zhang, Ce Zhang, Hao Huang, Yiran Li, Erqiang Hu, Gui Deng, Shanshan Guo, Yahui Wang, Weimin Li, Lina Chen

**Affiliations:** ^1^ College of Bioinformatics Science and Technology, Harbin Medical University, Harbin, Heilongjiang, China; ^2^ Department of internal medicine, Heilongjiang Commercial Hospital, Harbin, Heilongjiang, China; ^3^ Department of Cardiology, the First Affiliated Hospital of Harbin Medical University, Harbin, Heilongjiang, China

**Keywords:** RNA-Seq, canonical correlation analysis, co-expression network, ischemic cardiomyopathy, Pathology Section

## Abstract

Ischemic cardiomyopathy (ICM) is an important cause of heart failure, yet no ICM disease genes were stored in any public databases. Mutations of genes provided by RNA-Seq data could set a foundation for a variety of biological processes. This also made it possible to elucidate the mechanism and identify potential genes for ICM. In this paper, an integrated co-expression network was constructed using univariate and bivariate canonical correlation analysis for RNA-Seq data of human ICM samples. Three ICM-related modules were recognized after comparing between Pearson correlation coefficients of ICM samples and normal controls. Furthermore, 32 ICM potential genes were identified from ICM-related modules considering protein-protein interactions. Most of these genes were verified to be involved in ICM and diseases caused it by OMIM and literature. Our study could provide a novel perspective for potential gene identification and the pathogenesis for ICM and other complex diseases.

## INTRODUCTION

Ischemic cardiomyopathy (ICM) is an important cause of heart failure. It is a common type of dilated cardiomyopathy (DCM) results from coronary heart disease (CHD), according to National Heart, Lung, and Blood Institute, National Institutes of Health, U.S. Department of Health and Human Services. As other complex diseases, ICM is caused by interactions of genetic and environmental factors. Many studies have been conducted on ICM from different aspects, especially from the genome level [1, 2]. However, no ICM disease genes have been stored in public databases.

Nowadays, with the next-generation sequencing technology being widely used [3], studies for dysfuntions caused by gene mutations are still inadequate [4, 5]. Transcriptome data retrieved from next-generation sequencing data, especially RNA-Seq data, were superior to expression profiles from the microarray technology [6]. The high sensitivity of RNA-Seq data could provide accurate and precise expression data from various levels, such as exon, position and allelic levels [7].

Genes causing complex diseases always participate in common biological processes in various kinds of biological networks [8, 9]. Of all biological networks, co-expression networks could provide information of co-regulation genes that function in regulation processes and relationships of transcriptome components disturbed by environment. Therefore, co-expression networks could be used as effective tools to study gene functions, biological processes and complex disease mechanisms [10]. Traditionally, correlation coefficients between expression values of gene pairs were used to evaluate their relationships and construct co-expression networks. Each expression value was represented by a single variable without considering expression differences caused by exons, positions or allels [7, 11, 12]. On the contrary, canonical correlation analysis (CCA) could measure correlations between two sets of variables [13]. Thus, CCA could be used to examine complex patterns of gene expressions and accurately represent co-expression relationships by considering variations of transcripts. In co-expression networks, modules, or highly interconnected regions, are mutually correlated [14]. Genes in the same modules tend to have similar functions, or involve in common biological processes [15]. Comprehensive understanding of diseases could be provided by analyzing biological data based on network modules.

**Figure 1:**
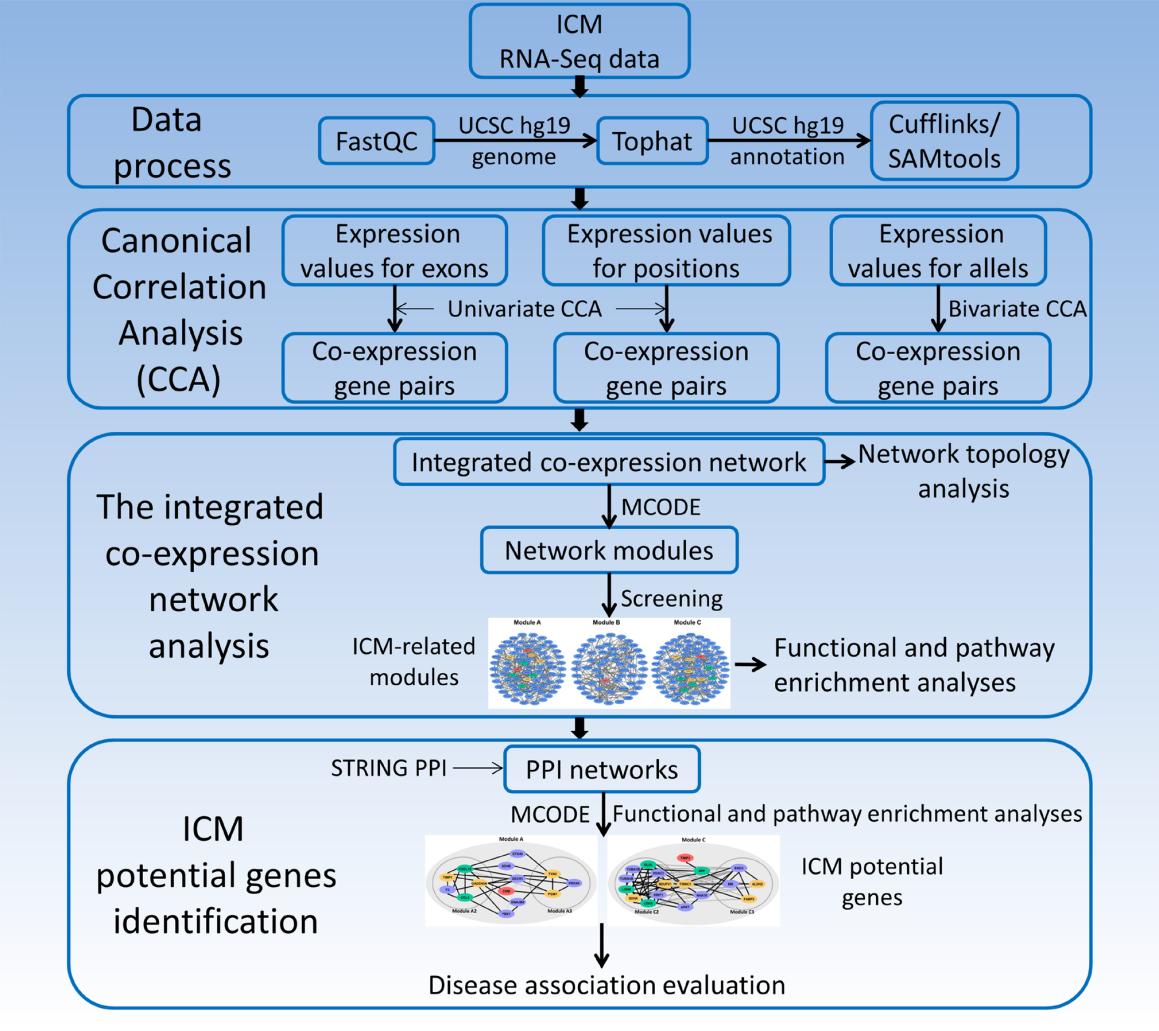
The identification of potential genes for human ischemic cardiomyopathy based on RNA-Seq data

Hence, to construct co-expression networks to investigate genes in ICM process comprehensively, co-expression gene pairs were identified using univariate and bivariate CCA. Here, CCA was performed for the expression data of the exon, position and allelic levels obtained from RNA-Seq data of human ICM samples. Modules and their functions were further analyzed. Since protein-protein interaction (PPI) could reflect functions and cooperation of proteins/genes [16], sub-modules from PPI networks could help to identify potential genes of ICM (Figure 1).

## RESULTS

### The integrated co-expression network

Co-expression gene pairs of ICM samples for expression data of exons and positions were identified using the univariate CCA, and those for expression data of ASE were identified using the bivariate CCA (see Materials and Methods). The integrated co-expression network was constructed by integrating 17045 co-expression gene pairs between 1135 genes with non-zero correlations from all three levels.

### Topological property of the integrated co-expression network

The assortativity of a biological network, a random network and the integrated co-expression network were measured by their assortativity coefficients (see Materials and Methods). It was showed that the biological network was disassortative (assortativity coefficient <0) as high degree nodes were more likely to connect to low degree nodes. For the random network, the assortativity coefficient was close to 0. These results were consistent with previous studies [6]. Our integrated co-expression network was disassortative since the assortativity coefficient<0 (Table 1). These results indicated that our integrated co-expression network had similar topology features as biological networks.

**Table 1:** Assortativity coefficients for various types of networks

Network type	Assortativity coefficient
Biological network	−0.2305
Random network	−0.0489
The integrated co-expression network	−0.1233

### ICM-related modules

**Figure 2:**
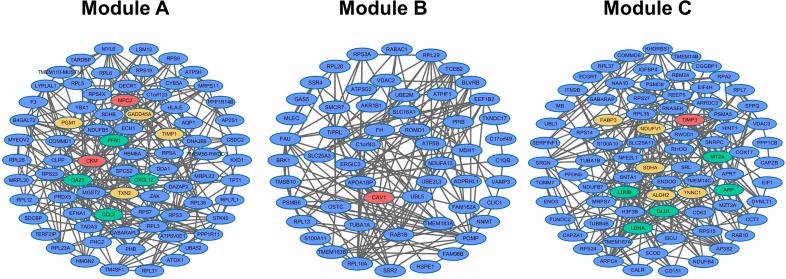
Three ICM-related modules (Module A, B and C) Red, yellow and green genes were verified to be related to ICM, DCM, and CHD, respectively.

Modules of the integrated co-expression network could reflect underlying pathological disease mechanisms. Here, network modules were detected using MCODE. After screening by comparing between Pearson correlation coefficients of ICM samples and normal controls (see Materials and Methods), 3 ICM-related modules were recognized and named as Module A, B and C (Figure 2).

**Figure 3:**
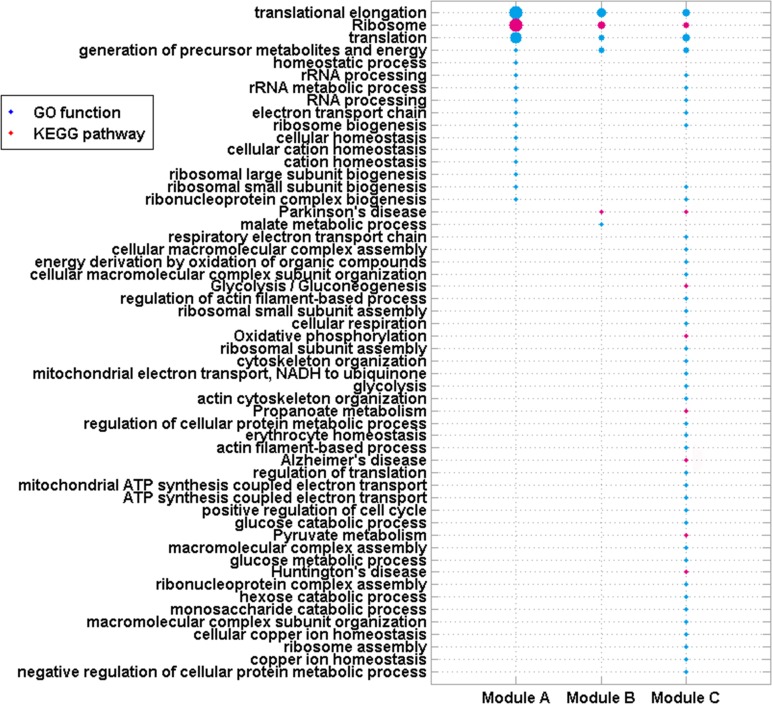
GO functions and KEGG pathways enriched by genes in 4 modules Node size is proportional to −log(P-Value) of each enriched function/pathway.

BP_Fat of GO and pathways of KEGG enriched for these 3 modules using DAVID were selected as stated in Materials and Methods (P-Value<0.05, Figure 3). All three modules were enriched in functions of “translation elongation”, “translation” and “generation of precursor metabolites and energy”, and the pathway of “Ribosome”. These enriched functions and pathways have been verified to be associated with ICM and diseases caused it (DCM and CHD).

The function of “generation of precursor metabolites and energy”, “electron transport chain” (enriched by Module A and C), the “Oxidative phosphorylation” pathway (enriched by Modules C) and other functions/pathways all result in the formation of energy. Moreover, mitochondrion is the site where all three modules were mainly localized, which indicated that energy was involved in ICM. It was showed that ICM and other cardiovascular diseases involved the misuse of energy and oxygen with high uptake and oxidation of fatty acids [17]. Moreover, ATP-binding cassette (ABC) B10 expression and heme levels were altered in hearts of patients with ICM. The mitochondrial transporter ABCB10 was reported to export heme out of the mitochondria. Heme plays a critical role in gas exchange, mitochondrial energy production, and antioxidant defense in cardiovascular system [18]. Alterations in energy-metabolism have also been detected in biopsies from patients with ICM [19].

Homeostasis is the process that living organisms use to regulate their internal equilibrium, which keeps the health of human bodies. Most human diseases, including cardiovascular diseases, involve the disruption of normal homeostasis [20]. ICM could be caused by dysfunction of such biological processes, e.g. “homeostatic process” and “cellular homeostasis” [1, 21].

“Ribosome” is the site where all three modules were mainly localized. It is the place of “translation”, which is the process that proteins are synthesized. During “translation elongation”, amino acids are brought to the ribosome, and joined to form proteins in the order specified by mRNAs. These biological processes/pathways were greatly associated with DCM and CHD. Mice with mutant mTOR rapidly developed DCM with cardiomyocyte growth defects resulted from impaired protein translation efficiency [22]. The Food and Drug Administration approved drug mipomersen could treat FH, one disease that could cause CHD, by inhibiting the apolipoprotein translation [23]. Androgen deficiency was shown to play a part in CHD and other cardiovascular diseases. These symptoms resulted from altered or damaged androgen synthesis, regulation or binding. In this process, genomic transcription and translation were also affected [24]. Mencarelli et al. found a different RNA secondary structure could change translation and protein synthesis, which was associated with CHD, type 2 diabetes and hypertension in the carriers [25].

These results exhibited the importance of ICM-related modules detected from the integrated co-expression network. These modules participated in many vital biological processes, which were associated with ICM.

### ICM potential genes

**Figure 4:**
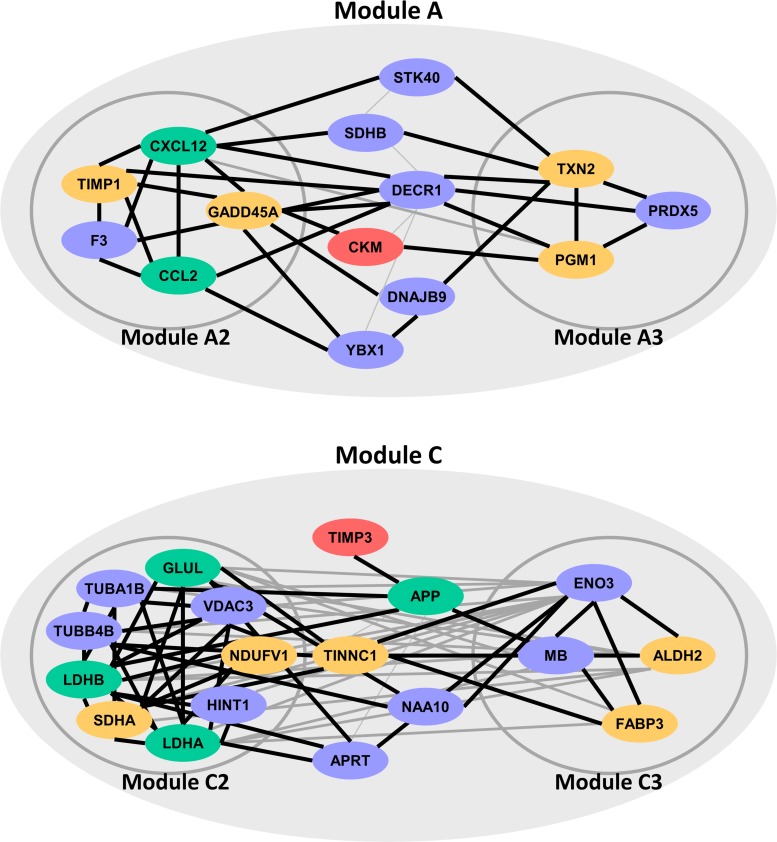
ICM potential genes locating in or mediating four sub-modules (Module A2, A3, C2 and C3) Red, yellow and green genes were verified to be related to ICM, DCM and CHD, respectively.

Three PPI networks were built for three ICM-related modules, respectively. Since genes in these PPI networks were enriched in biological processes associated with ICM, these PPI networks were also ICM-related. Using MCODE, 3, 2 and 6 sub-modules were recognized from PPI networks of Module A, B and C, respectively. These sub-modules were named as Module A1-A3, B1-B2, and C1-C6. Module A1, B1-B2, C1 and C4-C6 were mainly localized to important subcellular organelles, such as ribosome or mitochondrion. It was worth noting that Module A2, A3, C2 and C3 were significantly enriched in translation, energy or homeostasis-related biological processes. These biological processes were verified to be strongly associated with ICM and its two major causes, DCM and CHD. As a result, 32 genes locating in or mediating Module A2, A3, C2 and C3 could act as ICM potential genes (Figure 4).

Since ICM was caused by DCM and CHD and no ICM disease genes were stored in any public databases, the disease associations of these genes were evaluated by the Online Mendelian Inheritance in Man (OMIM, Updated 9 July 2016) database [26] and recent literature for ICM, DCM or CHD.

For genes in Module A2, the research of Bironaite et al. showed that TIMP1 significantly increased in sera in DCM myocardium [27]. Results from DNA methylation profile between DCM patients and normal individuals showed that selenium deficiency increased the expression of the Gadd45α, i.e. gene GADD45A [28]. CCL2 was CHD disease gene in OMIM [26]. Huang et al. and Franceschini et al. observed significant association between SNPs of CXCL12, rs1746048-C and rs501120, and an increased risk of CHD in Han Chinese and US cohorts, respectively [29, 30]. Many studies have shown that DCM was one of main clinical manifestations of PGM1 (in Module A3) deficiency [31, 32]. Western blot and histological analysis revealed that TXN2 (Module A3) protein expression levels were reduced in hearts from patients with DCM. Cardiac-specific TXN2 knockout mice developed DCM at 1 month of age with increased heart size, reduced ventricular wall thickness, and a progressive decline in left ventricular contractile function [33]. CKM in Module A that interacted with Module A2 and A3 was reported to be associated with ICM since protein levels of CKM were found to be reduced in ICM patients [34].

Gene SDHA of Module C2 was DCM disease gene stored in OMIM [26]. Ono et al. discovered that NDUFV1 of Module C2 was involved in the pathogenesis of DCM since NDUFV1 production decreased significantly in the myocardium of patients with DCM [35]. The rs10911021 SNP at the locus of gene GLUL of Module C2 has been associated with an increased risk of CHD in individuals with type 2 diabetes [36]. After 24 hours of treatment with lovastatin, which is widely used in prevention and treatment of CHD, the LDHA (in Module C2) mRNA levels went up. When the treatment time was extended to five days, the protein levels of LDHA were up-regulated, while LDHB (in Module C2) mRNA levels and protein levels were both down-regulated [37]. Sun et al. revealed that levels of ALDH2 in Module C3 were down-regulated in hearts from DCM patients [38]. Mean levels of FABP3 (Module C3) in DCM groups were significantly higher than in the control group in many researches. Thus, FABP3 was often used as a plasma biomarker in DCM, and played a significant role in its development [39, 40]. Moreover, TNNC1 of Module C interacting with both Module C2 and C3 were DCM disease genes stored in OMIM [26]. Though TIMP3 of Module C did not interact with Module C2 and C3 directly, it interacted with gene APP that interacted with both Module C2 and C3. APP has been indicated to be involved in CHD by several studies [41, 42]. TIMP3 has also been shown to be significantly down-regulated in patients with ICM [43].

After database and literature evaluation, 17 ICM potential genes were verified to be involved in ICM, DCM and CHD by OMIM and literature. For other ICM potential genes, though no disease association record was found in OMIM or literature, they also participated in ICM associated biological pathways. For example, SDHB in Module A and ENO3 in Module C3 were annotated to the process of “generation of precursor metabolites and energy”. “Cellular homeostasis” was annotated by gene PRDX5 of Module A3.

## DISCUSSION

RNA-Seq data from the next-generation sequencing technology could offer high-quality expression data of various levels for transcriptome analysis. Plenty of transcriptome information could be provided by expressions of exons, positions and allels [44], which should be considered comprehensively. Hence, in this paper, univariate and bivariate CCA was employed to identify co-expression gene pairs for the exon, position and allelic data from RNA-Seq data, respectively. The integrated co-expression network was constructed by aggregating three sets of co-expression gene pairs. This network was biologically significant and could be used as the context for exploring potential genes and the pathogenesis of diseases.

Additionally, three ICM-related modules were screened from the integrated co-expression network after comparing between Pearson correlation coefficients of ICM samples and normal controls. These modules could reflect alterations from normal to disease states. Therefore, they were significantly enriched in disease associated biological processes, such as translation, energy generation and homeostasis. This indicated that some important genes in these modules might take vital roles in the process of ICM.

To consider real interactions between genes in each ICM-related module, we built one PPI network for each ICM-related module. Genes in these PPI networks were enriched in many vital biological processes, such as “translation elongation”, “translation” and “generation of precursor metabolites and energy”, and the pathway of “Ribosome”, which were associated with ICM. Besides these common biological processes, these PPI networks could be enriched in different biological processes. These different biological processes were also ICM-related processes and could represent different aspects for ICM mechanism. Sub-modules from these PPI networks could provide better understanding of functions and cooperation for genes in ICM. 32 ICM potential genes locating in or mediating sub-modules were identified, which were significantly enriched in translation, energy or homeostasis-related biological processes. Of these ICM potential genes, 2 were verified to be involved in ICM, 9 in DCM, 6 in CHD by OMIM and literature, and others participated in ICM associated biological pathways.

In our method, relationships between genes were detected based on expression values of different levels, i.e. exon, position and allelic levels, from RNA-Seq data of ICM samples. Recently, some sequence analysis tools have been proposed, such as Pse-in-One, repRNA and repDNA [45–47]. The three tools could generate various vectors of important features once sequences of DNAs, RNAs or proteins were given. We speculate that co-regulation/function relationships between genes with sequence variations could be obtained from various vectors of important features generated by the three tools. With relationships from the three tools incorporated into our model, we hope that a much more powerful method considering sequence patterns could be developed in the future.

## MATERIALS AND METHODS

### Data

RNA-Seq data of human ICM samples, GSE48166, were obtained from the Gene Expression Omnibus (GEO) database (http://www.ncbi.nlm.nih.gov/geo/) [48], which contained 15 ICM and 15 normal samples. The reference human genome data were the hg19 file from the UCSC database (http://genome.ucsc.edu/) [49]. The human genome annotation data were also from the UCSC database (http://genome.ucsc.edu/cgi-bin/hgTables?command=start).

The expression values of exon, position and allelic levels were obtained after processing RNA-Seq data of 15 ICM samples using FastQC [50], Tophat [51], Cufflinks [52] and SAMtools [53]. Expression values of the exon or position level were read counts of exons or positions, and expression values of the allelic level, allel specific expressions (ASE), were read counts of allel pairs of single nucleotide polymorphisms (SNPs). The expression values of 5105 exons, 5280 positions and 5333 allel pairs from 15 ICM samples were obtained after data processing.

### Identification of co-expression gene pairs

In this paper, univariate and bivariate CCA were employed to identify co-expression gene pairs, which could detect the maximum correlation between two sets of variables [6].

### Univariate canonical correlation analysis

The univariate CCA was performed for the expression data of the exon or position level of two genes, *g*_1_ and *g*_2_. *g*_1_ had *p* exons or positions, and *g*_2_ had *q* exons or positions, *p* ≤ *q*. *X*_e_^(1)^ and *X*_e_^(2)^ were vectors of expression values for the *e* th exon or the *e* th position of *g*_1_ and *g*_2_ for ICM samples. Thus, the expression data of the exon or position level of these two genes could be represented as *X*^(1)^ = [*X*_1_^(1)^,…, *X*_p_^(1)^]*^T^* and *X*^(2)^ = [*X*_1_^(2)^,…, *X*_q_^(2)^]*^T^*. Linear combinations of exon or position expression values from two genes, *U* and *V*, were represented as *U* = *a^T^ X*^(1)^ and *V* = *b^T^ X*^(2)^, respectively. The maximum correlations between different pairs of *U* s and *V* s, *λ*_1_, *λ*_2_,.. *λ*_p_, *λ*_1_^2^≥ *λ*_2_^2^ ≥… ≥ *λ*_p_^2^ with the significance *p*_i_ = (*i* = 1, …, *p*) were calculated [13].

Since correlations with *p*_i_ ≤ 0.05 was significant, the final correlation, *w_exon/position_*, between two genes was defined as

$$\begin{array}{l} \;w_{\text{exon}/\text{position}}=\frac{\sum_{i=1}^{p}\lambda_{i}I\left(\log P_{i}\right)}{\sum_{i=1}^{p}I\left(\log P_{i}\right)}\\ \text{where }I\left(\log P_{i}\right)=\{\begin{array}{cc} 0 & P_{i}>0.05\\ -\log P_{i} & P_{i}\leq 0.05 \end{array}\cdot w_{\text{exon}/\text{position}}=0\text{ if }\sum_{i=1}^{p}I\left(\log P_{i}\right)=0. \end{array}$$

Gene pairs with non-zero *w_exon/position_* were then used to construct the integrated co-expression network [54].

### Bivariate canonical correlation analysis

The bivariate CCA was performed for the ASE data of SNPs from two genes, one with *s* SNPs, the other with *t* SNPs, *s* ≤ *t*. *Y*_i_^(1)^ and *Y_i_^(2)^* were vectors of the expression values for two allels of the *l* th SNP of one gene, and *Z*_l_^(1)^ and *Z*_l_*^(2)^* were vectors of the expression values for two allels of the *l* th SNP of one other gene for ICM samples. Thus, the ASE data of these two genes could be represented as *Y* = [*Y*_1_^(1)^,…, *Y*_s_^(1)^, *Y*_1_^(2)^,…, *Y*_t_^(2)^]*^T^* = [*Y*^(1)^,*Y*^(2)^]*^T^* and *Z* = [*Z*_1_^(1)^,…, *Z*_s_^(1)^, *Z*_1_^(2)^,…, *Z*_t_^(2)^]*^T^*= [*Z*^(1)^, *Z*^(2)^]*^T^*. Linear combinations of ASE from two genes, *M* and *N*, were represented as *M*=*c^T^Y* [*c*^(1)^]*^T^*,*Y*^(1)^+[*c*^(2)^]*^T^ Y*^(2)^ and *N*=*d^T^Z* [*d*^(1)^]*^T^*,*Z*^(1)^+[*d*^(2)^]*^T^ Z*^(2)^, respectively. The maximum correlations between different pairs of *M* s and *N* s, *γ*_1_, *γ*_2_,.. *γ*_p_, *γ*_1_^2^≥ *γ*_2_^2^ ≥… ≥ *γ*_p_^2^ with the significance *p_i_* were calculated [13].

Since correlations with *p_i_* ≤ 0.05 was significant, the final correlation between two genes *w_allel_* was defined as

$$\begin{array}{l} \;w_{\text{allel}}=\frac{\sum_{j=1}^{2s}\gamma_{j}I\left(\log P_{j}\right)}{\sum_{j=1}^{2s}I\left(\log P_{j}\right)}\\ \text{where }I\left(\log P_{j}\right)=\{\begin{array}{cc} 0 & P_{j}>0.05\\ -\log P_{j} & P_{j}\leq 0.05 \end{array}\cdot w_{\text{allel}}=0\text{ if }\sum_{i=1}^{2s}I\left(\log P_{j}\right)=0. \end{array}$$

Gene pairs with non-zero *w_allel_* were then used to construct the integrated co-expression network [54].

### Construction and analysis of the integrated co-expression network

Co-expression networks are undirected graphs, where nodes correspond to genes, and edges between genes represent co-expression relationships, i.e. correlations between co-expression gene pairs. The integrated co-expression network was constructed by integrating all co-expression gene pairs with non-zero correlations (*w_exon/position_* or *w_allel_*) for the expression data of the exon, position and allelic levels obtained from RNA-Seq data of human ICM samples.

### Topological analysis of the integrated co-expression network

The topological property of the integrated co-expression network was evaluated by assortativity. Assortativity is an important measure for network topology, which describes the tendency of a node connecting to similar nodes in a network. Assortativity coefficient was used to measure network assortativity by the Pearson correlation coefficient between degrees of connecting node pairs [55]. If the assortativity coefficient >0, the network was assortative; if the assortativity coefficient <0, the network was disassortative. To compare assortativity of different types of networks, a biological network and a random network were constructed. The nodes and edges of the biological network were from KEGG pathways, and those for the random network were selected randomly from KEGG pathways.

### Detection and function of ICM-related modules

Modules of co-expression network constructed from disease samples could be linked to a particular disease phenotype and help to uncover disease mechanisms [56]. MCODE was employed to detect network modules from the integrated co-expression network [57]. To further screen ICM-related modules, differences between Pearson correlation coefficients of expression values for ICM samples and those for normal controls from GSE48166 were calculated for each module. Then, the difference was compared with those of 1000 random modules, which were constructed by selecting the same number of genes as modules from MCODE. If the real difference was significantly greater than the random ones (permutation test, *p* < 0.05), the module was considered as ICM-related.

Functional and pathway enrichment analyses were performed for these screened ICM-related modules using the Database for Annotation, Visualization, and Integrated Discovery (DAVID, https://david.ncifcrf.gov/). To understand the significance of genes in the process of ICM, BP_Fat (biological process) of Gene Ontology (GO) [58] and pathways of Kyoto Encyclopedia of Genes and Genomes (KEGG) [59] with *P*-Value<0.05 were selected.

### Identification of ICM potential genes

It was necessary to consider real interactions between genes in ICM-related modules. PPIs of genes in each module were obtained from the STRING database (v10, http://string-db.org/) [60]. Three PPI networks were built for these modules, respectively. MCODE was employed to recognize sub-modules from these PPI networks, respectively. Functional and pathway enrichment analyses were also performed as aforementioned. Genes in sub-modules that were significantly enriched in biological processes associated with ICM (*P*-Value<0.05) could act as ICM potential genes. In addition, genes mediating this kind of sub-modules, i.e. interacting with sub-modules, could also be ICM potential genes.

## CONCLUSIONS

Though no ICM disease genes were stored in public databases, ICM-related modules screened from the integrated co-expression network constructed for ICM RNA-Seq data could provide more genomic and molecular information for biological processes and disease mechanisms. Taking PPIs into consideration, 32 genes locating in or mediating sub-modules were identified as ICM potential genes. 17 genes were verified to be involved in ICM, DCM and CHD by OMIM and literature. Our method will become an effective and powerful tool for identifying potential genes and elucidating the pathogenesis of complex diseases and their subtypes.
